# The First District Dental Society of New York

**Published:** 1886-11

**Authors:** 


### THE FIRST DISTRICT DENTAL SOCIETY OF NEW YORK.

REPORTED EXPRESSLY FOR THE INDEPENDENT PRACTITIONER.

A special meeting of the above named society was held at the rooms of The S. S. White Dental Manufacturing Co., on Tuesday, September 7, 1886, at 8 o'clock, P. M. On account of the absence of the President and Vice-President, Dr. W. W. Walker, Chairman of the Executive Committee, took the chair, and called for incidents of office practice.

Dr. Geo. F. Reese spoke at some length of his method of inserting amalgam fillings. He lines all cavities with oxy-phosphate of zinc, which he mixes rather thin, and inserts in the cavity. Before it sets he presses the amalgam over it, forcing out all surplus oxy-phosphate, leaving only a very thin layer of it. Dr. Reese claims to obtain better results, and no discoloration of the teeth. He inserts gold fillings in the same manner.

Dr. C. S. W. Baldwin said that he had used oxy-phosphate of zinc as a lining for cavities for many years, but becoming dissatisfied with the results, he had now entirely discarded it.

Dr. Wm. H. Atkinson then read the regular paper for the evening, his subject being "The Evolution of Preserving Teeth by Filling Materials and Methods."

He first described the state of the dental profession, the crude instruments in use when he entered it, and after a detailed description of its development up to the present time, he concluded his paper as follows:

"Whether we are nearing the end of improvement in filling teeth or not, we are at least progressing in the order of the inspirational necessities, from early practices through varied experiences of means and methods down to the summation of those in the Herbst method, by using the hand impact and rotation of point in combination, thus securing adaptation of filling to wall of cavities heretofore unattained by any method. The hand may rotate the unpolished end of the filler and effect the work in good shape; but rotation of point in the hand piece of the engine saves time and secures superiority of adaptation, besides being less unpleasant to the patient. In fact, in Herbst's own hand, the pressure of packing the gold was rather pleasurable than otherwise, in my own mouth.

"A glance at what has already been said will reveal the sum of a long and varied trial of means and methods resorted to in attempts to save decayed teeth by filling. No single partial presentment of method can compare with a combination of all the good points in the different methods conjoined in successive adaptation, which I regard the Herbst method as presenting."

Dr. G. A. Mills related some details of his experiences when entering upon dental practice. He described the crude instruments as well as methods at that time in use for filling teeth. He spoke of the wonderful improvement made since that time, and especially of the inventions of Dr. Herbst.

Dr. C. F. W. Bödecker thanked Dr. Atkinson for his interesting paper, and called attention to a letter published in the September number of the *Dental Cosmos*, in which it is stated that everything which Dr. Herbst claims as his invention had been in use in this country for many years. Dr. Bödecker affirmed that all which Dr. Herbst claimed is original with him, for he is not able to read English, nor does he attend the meetings of dental societies more than once or twice a year. About three years ago, when Dr. Bödecker was in Europe, Dr. Herbst told him of his invention of the steel pen device for holding the gum and rubber dam above the margin of cervical cavities while filling. He fancied that he had made a great discovery, and was very much surprised when told by Dr. Bödecker that there already were a number of special clamps for that purpose.

Dr. Bödecker admitted that many little devices like the one mentioned had been in use in this country for years, but asserted that he believed that the re-invention by Dr. Herbst was entirely original, and that they had not been copied, for the devices of Dr. Herbst indicate their originality. It was very strange that no one had practiced the Herbst method if, as it is claimed, it was known long ago. He advised the gentlemen who proposed to try the new method not to become discouraged, even though their first efforts were failures. It is easily comprehended when demonstrated by any competent operator. He further advised that beginners should not at first attempt to finish their work by the rotary method, but in the last layers use the mallet until experience had made them better acquainted with the manipulation. He thinks that the future will show very many American operators who will make better operations by the rotary method than can Herbst himself. He announced that he (Dr. Bödecker), would operate by the Herbst system at every clinic, and that he had several promises from other practitioners who are willing to operate by this method at the clinics.

Dr. Bödecker then spoke of the practice of lining cavities with oxy-phosphate of zinc, as mentioned by Dr. Reese. He followed this some years ago, but, like Dr. Baldwin, he had now discarded it. He commended the lining of cavities with gold, as demonstrated by Dr. Herbst at his clinics. He also stated that he had been using Herbst's amalgam lately, and found it excellent. Herbst told him that, according to his experience, the silver, and not the mercury in the amalgam, should be blamed for the contraction as well as for the discoloration.

Dr. Wm. H. Atkinson commended Dr. Bödecker for vindicating Herbst. He said that yesterday he filled a tooth by the Herbst process, and did exactly as Dr. Bödecker advised; he adjusted the last layer with the mallet. He felt that he could vouch for the honesty of Herbst.

Dr. W. G. A. Bonwill spoke at length concerning the condensing of gold by his mechanical mallet, with which he can pack it very fast, as he had demonstrated at the New Jersey State Society meeting, at Asbury Park. He thought that the patient's comfort should not so much be taken into consideration as the time demanded, and claimed that with the mechanical mallet he could insert a filling quicker than Herbst could by the rotary process. He asked Dr. Bödecker why Dr. Herbst used so much hand pressure for the introduction of his gold.

Dr. Bödecker said that Dr. Herbst stated at the meeting of the New Jersey Society, that "If he had been able to condense gold like Dr. Bonwill, he would never have felt the need of another method." There are very few operators who can insert gold as fast as Dr. Bonwill can, but about one-half of this time could be saved if two-thirds of the operation were made by the Herbst method, and the other third by the mallet. The last layers of gold take about as much or more time to put on by the rotary process, as when done by the mallet. Dr. B. further stated that the great amount of hand pressure spoken of by Dr. Bonwill was mostly used in the last layers, and in that particular Dr. Bonwill was right.

